# Antibiotic exposure and risk of overweight/obesity in children: a biomonitoring-based study from eastern Jiangsu, China

**DOI:** 10.3389/fpubh.2024.1494511

**Published:** 2024-11-08

**Authors:** Huamin Sun, Jianping Huang, Yijing Zhou, Xinying Guo, Man Jiao, Xingchen Zhu, Weiwei Tan, Weibing Zhang

**Affiliations:** ^1^Nantong Center for Disease Control and Prevention, Nantong, Jiangsu, China; ^2^Jiangsu Provincial Center for Disease Control and Prevention, Nanjing, Jiangsu, China

**Keywords:** antibiotics, urine, overweight, obesity, children

## Abstract

**Objective:**

To describe antibiotic exposure in children and explore its association with overweight/obesity.

**Methods:**

In June 2022, 328 kindergarten and primary school children were selected from Nantong city in Jiangsu Province. Questionnaires were distributed, and morning urine samples were obtained. Total urinary concentrations of 41 antibiotics were measured using ultra-performance liquid chromatography and tandem mass spectrometry. The rates of antibiotic exposure were expressed as percentages (%), specific percentiles (*P*_95_ and *P*_99_), and the maximum values were used to describe the concentration of antibiotics. The association between urinary antibiotic creatinine-adjusted and overweight/obesity was analyzed using logistic regression.

**Results:**

A total of 328 children were initially recruited, of which 295 aged 3–8 years met the inclusion criteria and were finally included in the study. The biomonitoring results revealed that 35 antibiotics were detected, with a total detection frequency of 98.31%. Among the included children, 24.75% were classified as overweight/obesity. Multinomial logistic regression analyses revealed significant associations between overweight/obese and exposure to veterinary antibiotics (VAs) and preferred veterinary antibiotics (PVAs). After adjusting for various overweight/obesity-relevant variables, higher exposure to sulfamethoxazole [OR = 2.35, 95% confidence interval (CI):1.17–4.70], norfloxacin (OR = 2.66, 95% CI: 1.01–7.08), and fluoroquinolones (OR = 1.97, 95% CI: 1.02–3.78) were significantly associated with overweight/obesity (*p* < 0.05). In addition, after stratification by sex and adjustment for confounding variables, sex-specific differences were observed in the association between antibiotic exposure and overweight/obesity. Notably, these associations were predominantly observed among boys.

**Conclusion:**

Children were extensively exposed to antibiotics. Exposure to certain types of veterinary antibiotics and preferred veterinary antibiotic exposure, mainly through food or drinking water, are associated with an increased risk of overweight/obesity in children.

## 1 Introduction

The incidence of childhood obesity is increasing annually and has become one of the most challenging global public health issues (1). Obesity in childhood not only seriously affects growth and development but also increases the risk of chronic obesity-related diseases in adulthood (2). The risk factors that lead to childhood obesity are extremely complex and include unhealthy dietary habits, lifestyle choices, and genetic factors. However, its pathogenesis remains unclear. Studies have shown that antibiotic exposure in early life may lead to intestinal microbiota imbalance and increase the risk of overweight/obesity in children (3, 4). Consequently, the relationship between antibiotic-altered microbiota and adiposity-related metabolic changes may contribute to the obesity in children.

In recent years, antibiotics have been increasingly used as growth promoters and disease control agents in animal husbandry and aquaculture (5, 6). Owing to the overuse of these antibiotics, an increasing number of antibiotic residues have been detected in soil, water, and animal-derived foods (chicken, beef, and milk) (7, 8). Humans can be exposed to antibiotics in two ways: through clinical or self-medication, leading to short-term high-dose exposure to human antibiotics, and through ingestion of contaminated food or drinking water, leading to long-term low-dose exposure to veterinary antibiotics (9, 10). Detecting the levels of antibiotics and their metabolites in urine can effectively reflect the level of human antibiotic exposure and provide a convenient and reliable method for assessing the body burden of antibiotics in the population (11). Previous research has shown that various antibiotics, especially veterinary antibiotics, are present in children’s urine (12, 13). This indicates that children are exposed to antibiotics extensively and for long durations, and compared to adults, children are more susceptible to the effects of antibiotics.

Epidemiological evidence suggests that antibiotic exposure is associated with childhood obesity (14–16). However, several studies have focused on school-age children, with fewer studies on preschoolers. In addition, these studies did not examine the relationship between antibiotic exposure from the daily diet or drinking water and childhood obesity. Therefore, this study aimed to monitor antibiotic exposure in children in Jiangsu, China and further evaluate the association between foodborne antibiotic exposure and childhood overweight/obesity.

## 2 Materials and methods

### 2.1 Study population

Between June and July 2022, two economically and geographically distinct areas in Nantong City were selected as study areas: Chongchuan (municipal district) and Rugao (prefecture-level city). Using a stratified sampling method, we randomly selected one kindergarten and one primary school from each area, followed by a random selection of small classes from each kindergarten and two classes in the first grade from each primary school. At least 75 children were selected from each of the four schools, resulting in 328 children. The inclusion criteria were as follows: (1) residing in the area for >6 months, (2) not in an acute state of morbidity, and (3) no antibiotic use in the past 30 days. Ultimately, we included 295 children aged 3–8 years (161 boys and 134 girls) who met the inclusion criteria. The study was approved by the Institutional Ethics Committee of the Nantong Center for Disease Control and Prevention, and consent forms were signed by the parents or guardians of the enrolled children.

### 2.2 Questionnaire survey, urine sampling, and anthropometric measurement

Potential confounding variables were assessed using a self-designed questionnaire completed by the guardians or parents of the children. Basic information collected included age, sex, school stage, location, duration of physical activity, and annual family income. The frequency of food consumption over the past 3 months was investigated using the Food Frequency Questionnaire (FFQ), which included vegetables, fruits, livestock meat products, poultry meat products, animal viscera, freshwater products, seafood, milk and dairy products, puffed food, and honey.

Morning urine samples were collected from the children using 30 mL polypropylene centrifuge tube. These samples were transported to the laboratory in an incubator containing ice packs and were stored at −80°C in the dark until analysis.

Body weight was measured to the nearest 0.1 kg and height to the nearest 0.1 cm using calibrated instruments and standard procedures by trained technicians. Body mass index (BMI) was calculated as weight in kilograms divided by height in square meters (kg/m^2^). Participants were classified as overweight/obese according to the criteria for children aged 2–18 in China as proposed by the Working Group on Obesity in China (WGOC) (17).

### 2.3 Antibiotic selection

Forty-one representative antibiotics were selected and classified into eight categories based on their mechanism of action: nine Sulfonamides, six Macrolides, eight *β*-Lactams, four Tetracyclines, nine fluoroquinolones, one Quinoxalines, one Lincosamides and three Chloramphenicols. These antibiotics were further grouped into four categories according to their usage: six human antibiotics (HAs), 14 veterinary antibiotics (VAs), five preferred human antibiotics (PHAs), and 16 preferred veterinary antibiotics (PVAs). Supplementary Table S1 shows the usage, chemical formula, molecular weight, and CAS number for each antibiotic.

### 2.4 Antibiotic analysis of urine samples

Urinary antibiotic levels were measured using ultra-performance liquid chromatography and tandem mass spectrometry (18). The detection limit (3S/N) of the method obtained using the LC-30A ultra-high-performance liquid chromatograph and the AB SCIEXQTRAP 4500 tandem mass spectrometer in the positive and negative ion modes was 0.01–1.25 μg·L^−1^. The recoveries of all analytes ranged from 61.3 to 128%, with the relative standard deviations (RDSs) (*n* = 5) of 1.2–14%.

To process the sample, 1.00 mL of urine was pipetted and mixed with 10 μL of mixed internal standard intermediate solution (1.0 μg/mL), followed by the addition of 0.30 mL of Na_2_ EDTA-Mcllvaine buffer (pH = 3.5). The mixture was vortexed for 30 s, and oscillated at 14,000 r/min for 5 min, and the supernatant was passed through the column. Methanol (3 mL) was used to wash the column, and the supernatant was collected after centrifugation and passed through the column again. The Oasis HLB solid-phase extraction column was sequentially activated with 3 mL of methanol, water (3 mL), and 3 mL of Na_2_ EDTA-Mcllvaine buffer. The supernatant was transferred throught the activated solid-phase extraction column at a flow rate of 1.0 mL/min. After evacuation using a vacuum pump, it was eluted with 3 mL of methanol at a rate of 1.0 mL/min. The eluate was collected, evaporated to near dryness under nitrogen, reconstituted to 0.5 mL with 10% methanol aqueous solution, vortexed to mix, passed through a 0.22 μm organic filter, and analyzed. For chromatographic analysis, a Kinetex^®^ F5 100A chromatographic column (50 × 3.0 mm, 2.6 μm) was used. The mobile phase consisted of 0.1% formic acid in water and 0.1% formic acid in acetonitrile, using gradient elution. Electrospray ionization was used with simultaneous scanning of positive and negative ions, and multiple reaction monitoring mode was employed for analysis. To correct the effect of urine dilution, we used the urine creatinine concentration to adjust the volume mass fraction of antibiotics. The corrected antibiotic creatinine concentration (μg/g creatinine) was calculated as the antibiotic concentration (ng/mL)/[urine creatinine concentration (mol/L) × 113.12 (g/mol)]. Urinary creatinine concentrations were measured using an automated biochemical analyzer [COBAS-8900, Roche Diagnostics (Shanghai) Co., Ltd., Shanghai, China].

### 2.5 Statistical analysis

The antibiotic concentrations below the limit of detection (LODs) were replaced with LOD s/2 to calculate the total detected concentration. The rates of antibiotic exposure were expressed as percentages (%), specific percentiles (P_95_ and P_99_), and the maximum values were used to describe the concentration of antibiotics. Children’s ages being skewed were represented by M (P_25_, P_75_). Comparison of rates between the two groups was performed using Pearson’s chi-square test. According to LODs and urinary creatinine-corrected concentrations of antibiotics, antibiotics are divided into three groups: <LODs (negative detection group), <median (low concentration group), ≥median (high concentration group). A total of 16 individual antibiotics were detected and > 10% of the samples were selected for further analysis. The association between urinary antibiotic creatinine-adjusted concentrations and overweight/obesity was analyzed using logistic regression. After stratification by sex, the association between different antibiotic exposure levels and overweight/obesity in children of different sexes was analyzed. All statistical analyses were conducted using SPSS software (version 21.0, IBM). A two-sided *p* < 0.05 was considered statistically significant.

## 3 Results

### 3.1 Characteristics of the study population

Among the 295 children aged 3–8 years, 73 were overweight/obese (39 were overweight and 34 were obese), resulting in a rate of overweight/obesity of 24.75%. The median age was 6.50 years (interquartile range: 4.00, 7.50). No statistically significant differences were observed in sex, school stage, location, physical activity time, annual family income, and consumption of vegetables, fruits, and livestock products between the normal-weight and overweight/obese groups. However, children with overweight/obesity showed a higher consumption of puffed food compared to children with normal weight (*p* < 0.05), while the consumption of meat products, poultry meat products, animal viscera, freshwater products, seafood, milk and dairy products, and honey were comparable between the two groups (Table 1).

**Table 1:** Demographic characteristics for participants of normal weight and overweight/obesity.

Variable	All (*n* = 295)	Normal weight (*n* = 222)	Overweight/Obesity (*n* = 73)	*p*-value
Sex, *n* (%)				
Boys	161	118(73.29)	43(26.71)	0.392
Girls	134	104(77.61)	30(22.39)	
School stages				
Kindergarten	141	106(75.18)	35(24.82)	0.977
Primary school	154	116(75.32)	38(24.68)	
Location, *n* (%)				0.758
Urban	142	108(76.06)	34(23.94)	
Suburb or rural	153	114(74.51)	39(25.49)	
Physical activity time				
≤3 h/day	209	154(73.68)	55(26.32)	0.330
>3 h/day	86	68(79.07)	18(20.93)	
Family annual income				
<10,000 yuan	29	24(82.76)	5(18.75)	0.615
10,000–24,999 yuan	113	84(74.34)	29(25.66)	
≥25,000 yuan	153	114(74.51)	39(25.49)	
Consumption of vegetables				
<Once/day	39	31(79.49)	8(20.51)	0.511
≥Once/day	256	191(74.61)	65(25.39)	
Consumption of fruits				
<Once/day	80	59(73.75)	21(26.25)	0.715
≥Once/day	215	163(75.81)	52(24.19)	
Consumption of livestock meat products				
<30 times/month	146	111(76.03)	35(23.97)	0.761
≥30 times/month	149	111(74.50)	38(25.50)	
Consumption of poultry meat products				
<30 times/month	65	52(80.00)	13(20.00)	0.315
≥30 times/month	230	170(73.91)	60(26.09)	
Consumption of animal viscera				
<Once/week	182	135(74.18)	47(25.82)	0.586
≥Once/week	113	87(76.99)	26(23.01)	
Consumption of fresh water products				
< Once/week	80	60(75.00)	20(25.00)	0.951
≥ Once/week	215	162(75.35)	53(24.65)	
Consumption of seafood				
< Once/week	106	79(74.53)	27(25.47)	0.990
≥ Once/week	189	143(75.66)	46(24.34)	
Consumption of milk and dairy products				
< Once/day	58	49(84.48)	9(15.52)	0.069
≥ Once/day	237	173(73.00)	64(27.00)	
Consumption of puffed food				
<3 times/week	225	178(79.11)	47(20.89)	0.006
≥3 times/week	70	44(62.86)	26(37.14)	
Consumption of honey				0.774
<Once/month	190	144(75.79)	46(24.21)	
≥Once/month	105	78(74.29)	27(25.71)	

### 3.2 Urinary concentrations of antibiotics

All 35 antibiotics were detected in urine, with a total detection frequency of 98.31%. Regarding antibiotic categories, sulfonamides exhibited the highest detection rates (81.69%), followed by fluoroquinolones (64.07%), macrolides (48.47%), *β*-lactams (43.39%), tetracyclines (25.42%), lincosamides (8.81%), chloramphenicols (3.73%), quinoxalines (0.00%). The detection rates of individual antibiotics ranged from 0.00 to 49.49%. Among them, 16 antibiotics, including sulfameter (49.49%), sulfaclozine (29.49%), sulfamerazine (38.98%), sulfamonomethoxine (28.81%), erythromycin (38.31%), azithromycin (21.02%), cefquinome (15.25%), amoxicillin (23.73%), oxytetracycline (16.27%), pefloxacin (18.98%), lomefloxacin (16.61%), danofloxacine (13.22%), sarafloxacin (15.25%), ofloxacin (12.88%), ciprofloxacin (10.17%), norfloxacin (12.20%), were detected in more than 10% of the participants. HAs, VAs, PHAs, and PVAs were detected in 26.10, 72.20, 55.25, and 90.17% of the urine samples, respectively. Moreover, two or more antibiotics were simultaneously detected in 93.90% of urine samples. Urinary concentrations ranged from <0 ng/mL to 14.9 μg/mL. The urinary concentrations of sulfaclozine and norfloxacin in children who were overweight/obese were significantly higher than those in normal-weight group (*p* < 0.05). Conversely, the urinary concentrations of sulfamerazine in children who were overweight/obese were significantly lower than those in normal-weight group (*p* < 0.05) (Table 2).

**Table 2:** Detection frequency and urinary concentration (ng/mL) distribution of included antibiotics in normal weight and overweight/obesity.

Antibiotics	Usage	All (*n* = 295)	Normal weight (*n* = 222)				Overweight/Obesity (*n* = 73)				*P*-value
		*n* (%)^a^	*n* (%)^a^	Percentile			*n* (%)^a^	Percentile			
				95%	99%	Maximum		95%	99%	Maximum	
Sulfonamides^b^		241(81.69)	178(80.18)	9.22	22.76	75.35	63(86.30)	8.49	11.32	12.02	0.241
Sulfamethoxazole	PVA	7(2.37)	4(1.80)	-	0.32	0.61	3(4.11)	0.08	0.39	0.47	0.370
Sulfameter	PVA	146(49.49)	106(47.75)	2.49	5.99	8.49	40(54.79)	4.35	7.37	8.13	0.296
Sulfaquinoxaline	VA	12 (4.07)	11(4.95)	0.08	0.20	0.34	1(1.37)	–	0.15	0.19	0.305
Sulfaclozine	VA	87(29.49)	58(26.13)	1.84	3.80	5.15	29(39.73)	2.46	3.45	3.70	0.027
Trimethoprim	PVA	16(5.42)	11(4.95)	0.09	12.65	75.35	5(6.85)	1.82	6.29	7.41	0.555
Sulfamerazine	PVA	13(4.41)	13(5.86)	0.69	16.17	20.83	0(0.00)	–	–	–	0.043
Sulfamerazine	PVA	115(38.98)	82(36.94)	4.60	8.24	10.10	33(45.21)	3.53	4.03	4.15	0.209
sulfachloropyridazine	VA	28(9.49)	17(7.66)	0.87	2.17	3.71	11(15.07)	1.46	4.50	5.26	0.061
Sulfamonomethoxine	VA	85(28.81)	65(29.28)	0.65	2.07	3.26	20(27.40)	1.35	3.22	3.69	0.758
Macrolides^b^		143(48.47)	107(48.20)	6.58	450.87	1434.50	36(49.32)	269.29	1,028.95	1,218.86	0.868
Erythromycin	PHA	113(38.31)	83(37.39)	1.96	13.66	35.14	30(41.10)	1.51	3.12	3.52	0.572
Clarithromycin	HA	1(0.34)	0(0.00)	–	–	–	1(1.37)	–	0.04	0.05	0.247
Azithromycin	HA	62(21.02)	48(21.62)	2.63	450.71	1,434.50	14(19.18)	268.06	1,028.70	1,218.86	0.657
Roxithromycin	HA	0(0.00)	0(0.00)	–	–	–	0(0.00)	–	–	–	–
Tilmicosin	VA	0(0.00)	0(0.00)	–	–	–	0(0.00)	–	–	–	–
Tylosin	VA	0(0.00)	0(0.00)	–	–	–	0(0.00)	–	–	–	–
β-lactams^b^		128(43.39)	98(44.14)	49.87	9,720.57	12349.12	30(41.10)	44.19	11922.34	1,4891.07	0.648
Ceftiofur	VA	2(0.68)	2(0.90)	-	0.19	0.27	0(0.00)	–	–	–	1.000
Cefquinome	VA	45(15.25)	37(16.67)	0.38	0.81	0.81	8(10.96)	0.39	1.25	1.46	0.239
Ampicillin	PHA	9(3.05)	8(3.60)	–	1.11	595.02	1(1.37)	–	6.70	8.38	0.460
cefotaxime	HA	12(4.06)	9(4.05)	–	1250.30	10017.91	3(4.11)	0.55	4.12	5.01	1.000
Penicillin V	PHA	6(2.03)	6(2.70)	–	0.25	0.67	0(0.00)	–	–	–	0.342
Penicillin V	PHA	11(3.73)	8(3.60)	–	1.63	2.17	3(4.11)	0.11	0.64	0.77	0.737
Amoxicillin	PHA	70(23.73)	51(22.97)	13.26	7169.05	9912.52	19(26.03)	42.87	11921.43	14891.07	0.595
Cefdinir	HA	13(4.4)	10(4.50)	0.00	61.15	2331.20	3(4.11)	0.23	0.98	1.17	1.000
Tetracyclines^b^		75(25.42)	52(23.42)	1.29	3.77	5.99	23(31.51)	1.31	1.62	1.70	0.169
Oxytetracycline	PVA	48(16.27)	32(14.41)	0.20	3.50	5.65	16(21.92)	0.27	0.39	0.42	0.132
Chlorotetracycline	PVA	5(1.69)	5(2.25)	–	0.84	1.10	0(0.00)	–	–	–	0.338
Tetracycline	PVA	12(4.07)	8(3.60)	–	0.33	0.55	4(5.48)	0.25	0.36	0.39	0.499
Deoxytetracycline	PVA	20(6.78)	14(6.31)	0.41	1.68	2.17	6(8.22)	1.20	1.46	1.52	0.594
Fluoroquinolones^b^		189(64.07)	136(61.26)	2.89	51.35	630.82	53(72.60)	3.69	5.02	5.35	0.080
Pefloxacin	PVA	56(18.98)	42(18.92)	1.19	2.32	15.75	14(19.18)	1.26	3.40	3.93	0.961
Lomefloxacin	PVA	49(16.61)	37(16.67)	0.45	0.55	0.62	12(16.44)	0.45	0.61	0.65	0.964
Danofloxacine	VA	39(13.22)	30(13.51)	0.90	1.58	2.08	9(12.33)	1.21	1.67	1.79	1.000
Sarafloxacin	VA	45(15.25)	30(13.51)	0.57	0.98	1.69	15(20.55)	0.74	1.17	1.28	0.188
Ofloxacin	PVA	38(12.88)	26(11.71)	0.62	1.09	1.87	12(16.44)	1.33	2.69	3.03	0.316
Difloxacin	VA	4(1.36)	4(1.80)	–	0.58	1.15	0(0.00)	–	–	–	0.575
Enrofloxacin	VA	15(5.08)	12(5.41)	0.15	1.26	1.81	3(4.11)	0.05	0.40	0.49	1.000
Ciprofloxacin	PVA	30(10.17)	23(10.36)	1.52	50.61	615.08	7(9.59)	0.92	2.81	3.28	1.000
Norfloxacin	PVA	36(12.20)	22(9.91)	0.47	0.88	0.95	14(19.18)	1.00	2.72	3.15	0.041
Quinoxalines^b^		0(0.00)	0(0.00)	–	–	–	0(0.00)	–	–	–	–
Quinocetone	VA	0(0.00)	0(0.00)	–	–	–	0(0.00)	–	–	–	–
Lincosamides^b^		26(8.81)	19(8.56)	0.19	1.39	9.17	7(9.59)	0.21	0.61	0.71	0.813
Lincomycin	PVA	26(8.81)	19(8.56)	0.19	1.99	9.17	7(9.59)	0.21	0.61	0.71	0.813
Chloramphenicols^b^		11(3.73)	8(3.60)	–	4.48	9.86	3(4.11)	0.75	6.66	8.14	0.071
Chloramphenicol	HA	0(0.00)	0(0.00)	–	–	–	0(0.00)	–	–	–	–
Thiamphenicol	PVA	6(2.03)	5(5.25)	–	1.87	3.70	1(1.37)	–	6.51	8.14	1.000
Florfenicol	VA	5(1.69)	3(1.35)	–	4.48	9.86	2(2.74)	–	3.53	4.41	0.600
HAs		77(26.10)	59(26.58)	38.32	1,535.35	12,349.12	18(24.66)	268.06	1,028.70	1,218.86	0.746
VAs		213(72.20)	160(72.07)	9.95	7.90	10.29	53(72.60)	4.67	5.17	5.29	0.930
PHAs		163(55.25)	123(55.41)	16.44	7170.01	9915.28	40(54.79)	43.67	11922.15	14891.77	0.927
PVAs		266(90.17)	197(88.74)	9.95	72.22	631.12	69(94.52)	8.43	10.20	10.64	0.150
HAs + PHAs		189(64.07)	144(64.86)	197.43	9721.46	12349.12	45(61.64)	515.83	12017.17	14892.51	0.619
VAs + PVAs		284(96.27)	212(95.50)	11.12	72.31	631.33	72(98.63)	11.16	13.59	14.19	0.384
All antibiotics^c^		290(98.31)	217(97.75)	199.37	9,722.88	12,350.73	73(100.00)	522.06	120,024.24	14,899.79	0.338

–, Below limits of detection (LODs). ^a^Positive detection (detection frequency,%). ^b^Total mass concentration of antibiotics in corresponding category. ^c^Sum of the mass concentrations of all antibiotics. HAs + PHAs was the sum of HAs and PHAs, and VAs + PVAs was the sum of VAs and PVAs.

### 3.3 The association between antibiotic exposure levels and overweight/obesity

A total of 16 individual antibiotics detected in >10% of the samples were selected for further analysis. Univariate conditional logistic regression analysis revealed that high urinary concentrations of ulfaclozine, norfloxacin, and fluoroquinolones were significantly associated with an increased risk of childhood overweight/obesity (*p* < 0.05). After adjusting for all covariates, we observed that the association between high exposure levels of sulfaclozine (OR = 2.35; 95% CI: 1.17–4.70), norfloxacin (OR = 2.66; 95% CI: 1.01–7.08), fluoroquinolones (OR = 1.97; 95% CI: 1.02–3.78) and an increased risk of childhood overweight/obesity remained significant (*p* < 0.05) (Table 3).

**Table 3:** Logistic regression analysis of association between antibiotic exposure levels and overweight/obesity in children (*N* = 295).

Antibiotics	Usage	Unadjusted^a^			Adjusted^b^		
		<LOD^c^	<Median^d^	≥Median^e^	<LOD^c^	<Median^d^	≥Median^e^
Sulfameter	PVA	Ref	1.33(0.70, 2.52)	1.33(0.70, 2.52)	Ref	1.32(0.69, 2.52)	1.33(0.69, 2.56)
Sulfaclozine	VA	Ref	1.44(0.69, 3.04)	2.34(1.18, 4.69)^*^	Ref	1.41(0.67, 3.00)	2.35(1.17, 4.70)^*^
Sulfamerazine	PVA	Ref	1.49(0.76, 2.90)	1.33(0.68, 2.62)	Ref	1.49(0.76, 2.91)	1.32(0.67, 2.59)
Sulfamonomethoxine	VA	Ref	0.81(0.36, 1.80)	1.02(0.48, 2.16)	Ref	0.79(0.36, 1.77)	1.00(0.47, 2.13)
Erythromycin	PHA	Ref	1.41(0.73, 2.74)	0.96(0.47, 1.94)	Ref	1.44(0.74, 2.81)	0.98(0.48, 2.00)
Azithromycin	HA	Ref	0.57(0.21, 1.54)	1.21(0.53, 2.77)	Ref	0.57(0.21, 1.55)	1.25(0.54, 2.91)
Cefquinome	VA	Ref	0.45(0.13, 1.57)	0.79(0.28, 2.22)	Ref	0.44(0.12, 1.52)	0.77(0.28, 2.18)
Amoxicillin	PHA	Ref	0.79(0.33, 1.91)	1.65(0.77, 3.54)	Ref	0.81(0.33, 1.96)	1.72(0.80, 3.71)
Oxytetracycline	PVA	Ref	2.00(0.83, 4.81)	1.37(0.54, 3.47)	Ref	2.00(0.83, 4.83)	1.36(0.54, 3.45)
Pefloxacin	PVA	Ref	0.83(0.32, 2.15)	1.22(0.51, 2.92)	Ref	0.84(0.32, 2.18)	1.19(0.50, 2.86)
Lomefloxacin	PVA	Ref	1.01(0.38, 2.66)	0.96(0.37, 2.51)	Ref	1.02(0.39, 2.70)	0.93(0.35, 2.48)
Danofloxacine	VA	Ref	0.80(0.26, 2.50)	1.00(0.35, 2.86)	Ref	0.83(0.26, 2.60)	0.96(0.33, 2.76)
Sarafloxacin	VA	Ref	1.89(0.76, 4.73)	1.45(0.57, 3.69)	Ref	1.90(0.76, 4.76)	1.44(0.56, 3.67)
Ofloxacin	PVA	Ref	1.15(0.40, 3.32)	1.87(0.71, 4.97)	Ref	1.14(0.39, 3.30)	1.94(0.73, 5.17)
Ciprofloxacin	PVA	Ref	0.75(0.21, 2.75)	1.10(0.34, 3.56)	Ref	0.73(0.20, 2.69)	1.15(0.35, 3.77)
Norfloxacin	PVA	Ref	1.70(0.61, 4.71)	2.71(1.02, 7.18)^*^	Ref	1.71(0.61, 4.76)	2.66(1.01, 7.08)^*^
Sulfonamides		Ref	1.09(0.48, 2.47)	2.12(0.97, 4.65)	Ref	1.08(0.48, 2.45)	2.10(0.96, 4.61)
Macrolides		Ref	1.11(0.59, 2.12)	0.98(0.51, 1.89)	Ref	1.14(0.60, 2.16)	1.01(0.52, 1.96)
β-lactams		Ref	0.60(0.29, 1.25)	1.22(0.64, 2.31)	Ref	0.59(0.28, 1.24)	1.24(0.65, 2.36)
Tetracyclines		Ref	1.84(0.87, 3.88)	1.21(0.55, 2.67)	Ref	1.81(0.85, 3.85)	1.21(0.55, 2.66)
Fluoroquinolones		Ref	1.39(0.71, 2.74)	1.99(1.04, 3.81)^*^	Ref	1.39(0.71, 2.75)	1.97(1.02, 3.78)^*^
Lincosamides		Ref	1.92(0.61, 6.08)	0.56(0.12, 2.59)	Ref	1.93(0.61, 6.11)	0.57(0.12, 2.65)
HAs		Ref	0.45(0.17, 1.21)	1.48(0.71, 3.08)	Ref	0.46(0.17, 1.23)	1.51(0.71, 3.20)
VAs		Ref	0.80(0.40, 1.60)	1.28(0.67, 2.47)	Ref	0.79(0.40, 1.58)	1.27(0.66, 2.44)
PHAs		Ref	0.79(0.40, 1.52)	1.19(0.64, 2.22)	Ref	0.80(0.41, 1.57)	1.22(0.65, 2.29)
PVAs		Ref	1.74(0.56, 5.41)	2.69(0.88, 8.23)	Ref	1.76(0.57, 5.47)	2.68(0.87, 8.24)
HAs+PHAs		Ref	0.71(0.36, 1.37)	1.05(0.56, 1.96)	Ref	0.72(0.37, 1.41)	1.09(0.58, 2.04)
VAs + PVAs		Ref	2.5(0.32, 20.87)	4.3(0.54, 35.00)	Ref	2.54(0.31, 20.70)	4.28(0.53, 34.51)

*P < 0.05. ^a^Univariable logistic regression. ^b^Multivariable logistic regression, covariates included were: consumption of puffed food. ^c^Below limits of detection (LODs). ^d^Below median levels among positive detected samples. ^e^Above median levels among positive detected samples. HAs + PHAs was the sum of HAs and PHAs, and VAs + PVAs was the sum of VAs and PVAs.

Sex-stratified analysis revealed that high exposure levels of sulfaclozine (OR = 3.97; 95% CI: 1.63–9.62), norfloxacin (OR = 5.40; 95% CI: 1.37–21.22), fluoroquinolones (OR = 2.60; 95% CI: 1.10–6.13) and low exposure levels of oxytetracycline (OR = 4.77; 95%CI:1.26–18.06), tetracyclines (OR = 4.49; 95 CI:1.53–13.17) were significantly associated with the risk of BMI-based overweight/obesity in boys (*p* < 0.05). In addition, the sex-stratified analysis revealed a significant positive association between high exposure levels of *β*-lactams (OR = 2.99; 95% CI: 1.08–8.32) exposure and BMI-based overweight/obesity risk in girls (*p* < 0.05) (Table 4).

**Table 4:** Logistic regression analysis of association between antibiotic exposure levels and overweight/obesity in different gender Children (*N* = 295).

Antibiotics	Usage	Boys^a^			Girls^a^		
		<LOD^b^	<Median^c^	≥Median^d^	<LOD^b^	<Median^c^	≥Median^d^
Sulfameter	PVA	Ref	1.01(0.44, 2.31)	0.97(0.41, 2.29)	Ref	1.76(0.57, 5.37)	2.04(0.67, 6.21)
Sulfaclozine	VA	Ref	1.65(0.60, 4.50)	3.97(1.63, 9.62)^*^	Ref	1.16(0.36, 3.74)	0.83(0.21, 3.28)
Sulfamerazine	PVA	Ref	1.00(0.41, 2.44)	1.29(0.53, 3.11)	Ref	2.51(0.87, 7.20)	1.71(0.56, 5.23)
Sulfamonomethoxine	VA	Ref	1.20(0.42, 3.43)	1.53(0.63, 3.69)	Ref	0.55(0.14, 2.08)	0.20(0.024, 1.72)
Erythromycin	PHA	Ref	1.08(0.46, 2.57)	0.97(0.38, 2.49)	Ref	2.51(0.83, 7.57)	1.06(0.34, 3.37)
Azithromycin	HA	Ref	0.70(0.18, 2.68)	1.18(0.33, 4.22)	Ref	0.57(0.12, 2.80)	1.55(0.47, 5.11)
Cefquinome	VA	Ref	0.40(0.09, 1.90)	0.66(0.17, 2.49)	Ref	0.48(0.05, 4.27)	1.15(0.22, 6.16)
Amoxicillin	PHA	Ref	0.79(0.22, 2.87)	1.14(0.35, 3.71)	Ref	0.77(0.21, 2.82)	2.26(0.61, 8.34)
Oxytetracycline	PVA	Ref	4.77(1.26, 18.06)^*^	3.23(0.96, 10.80)	Ref	0.88(0.22, 3.55)	0.26(0.03, 2.21)
Pefloxacin	PVA	Ref	1.73(0.53, 5.67)	1.44(0.46, 4.52)	Ref	0.23(0.03, 1.85)	0.78(0.19, 3.25)
Lomefloxacin	PVA	Ref	0.63(0.17, 2.42)	0.65(0.17, 2.51)	Ref	2.06(0.47, 9.05)	1.67(0.37, 7.44)
Danofloxacine	VA	Ref	0.27(0.33, 2.27)	0.75(0.19, 2.88)	Ref	1.84(0.42, 7.98)	1.32(0.23, 7.59)
Sarafloxacin	VA	Ref	2.55(0.73, 8.93)	2.10(0.69, 6.40)	Ref	1.60(0.38, 6.79)	0.63(0.07, 5.40)
Ofloxacin	PVA	Ref	1.51(0.43, 5.34)	6.04(1.40, 26.01)	Ref	0.49(0.05, 4.48)	0.44(0.05, 3.77)
Ciprofloxacin	PVA	Ref	1.01(0.19, 5.44)	1.22(0.21, 6.99)	Ref	0.89(0.10, 7.99)	1.26(0.24, 6.57)
Norfloxacin	PVA	Ref	1.86(0.42, 8.27)	5.40(1.37, 21.22)^*^	Ref	1.79(0.41, 7.79)	1.52(0.27, 8.40)
Sulfonamides		Ref	1.53(0.49, 4.80)	2.46(0.83, 7.24)	Ref	0.75(0.22, 2.53)	1.81(0.54, 6.02)
Macrolides		Ref	1.22(0.53, 2.80)	0.93(0.38, 2.29)	Ref	1.45(0.49, 4.34)	1.35(0.47, 3.85)
β-lactams		Ref	0.56(0.23, 1.37)	0.72(0.30, 1.75)	Ref	0.62(0.16, 2.42)	2.99(1.08, 8.32)^*^
Tetracyclines		Ref	4.49(1.53, 13.17)^*^	1.34(0.47, 3.82)	Ref	0.54(0.14, 2.14)	1.16(0.33, 4.03)
Fluoroquinolones		Ref	1.57(0.64, 3.83)	2.60(1.10, 6.13)^*^	Ref	1.21(0.41, 3.61)	1.54(0.54, 4.39)
Lincosamides		Ref	2.67(0.51, 13.94)	0.42(0.05, 3.63)	Ref	1.62(0.28, 9.28)	0.81(0.09, 7.60)
HAs		Ref	0.44(0.09, 2.06)	1.47(0.56, 3.85)	Ref	0.66(0.17, 2.51)	1.96(0.56, 6.91)
VAs		Ref	1.06(0.41, 2.72)	2.10(0.87, 5.09)	Ref	0.52(0.18, 1.49)	0.62(0.22, 1.77)
PHAs		Ref	0.60(0.26, 1.41)	0.85(0.36, 1.97)	Ref	1.11(0.36, 3.42)	2.23(0.82, 6.08)
PVAs		Ref	1.18(0.23, 6.05)	2.78(0.55, 14.01)	Ref	2.66(0.52, 13.55)	2.35(0.46, 11.98)
HAs+PHAs		Ref	0.76(0.33, 1.73)	0.69(0.29, 1.63)	Ref	0.71(0.21, 2.40)	2.43(0.86, 6.86)
VAs + PVAs		Ref	–	–	Ref	1.27(0.13, 12.20)	1.28(0.13, 12.56)

*p < 0.05. ^a^Multivariable logistic regression, covariates included were:consumption of puffed food. ^b^Below limits of detection (LODs). ^c^Below median levels among positive detected samples. ^d^Above median levels among positive detected samples. HAs + PHAs was the sum of HAs and PHAs, and VAs + PVAs was the sum of VAs and PVAs.

## 4 Discussion

In this population-based cross-sectional study conducted in the eastern Jiangsu Province, we monitored urinary exposure to 41 antibiotics in children and investigated the relationship between urinary antibiotic levels and the risk of overweight/obesity. Our findings revealed that children who were overweight/obese had higher exposure to VAs and PVAs (such as sulfaclozine, norfloxacin, and fluoroquinolones) compared to children with normal weight. However, no significant association was observed between HAs and PHAs antibiotic exposure and overweight/obesity risk in children. After stratifying by sex and adjusting for confounding variables, we identified sex-specific differences in the association between antibiotic exposure and overweight/obesity. In recent years, VAs and PVAs have been widely used as growth promoters in animal husbandry and aquaculture, resulting in antibiotic residues in animal-derived foods becoming the primary source of foodborne antibiotics for human consumption (19, 20). The respondents included in this study were healthy and had not used antibiotics clinically or self-medicated in the past month. Therefore, children are exposed to VAs and PVAs in their daily lives, mainly through contaminated food or drinking water. When the dietary habits and living environment contributing to antibiotic exposure in children remain unchanged, the concentration of antibiotics detected in a single urine test can represent the level of exposure to foodborne antibiotics in children over a certain period.

Our study included 41 antibiotics, with a detection rate of 98.31%. This finding suggests that children are being extensively exposed to antibiotics. Among the eight major categories of antibiotics, sulfonamides and fluoroquinolones showed the highest detection rates. However, these antibiotics are not commonly prescribed for children in clinical practice. Therefore, their high detection in urine samples is more likely due to residues of veterinary drugs in food or environment exposure to antibiotics (21). Compared to previous studies, our study revealed differences in antibiotic detection rates. For example, one study in Jiangsu found a 38.6% detection rate for seven antibiotics in children’s urine (22), while another study in Shanghai identified a detection rate of 56.0% for 15 antibiotics and two metabolites in children’s urine (23). The variation in detection rate can be attributed to the different antibiotics monitored in the study. This study monitored 41 antibiotics, which may have led to a higher detection rate than that reported in other studies. In addition, the detection rates of florfenicol and trimethoprim in urine samples from children in Shanghai (13), Shandong Province, and Guangdong Province (16) were higher than those reported in our study. These results suggest regional disparities in antibiotic exposure.

Current research suggests that the biological mechanism by which antibiotics promote lipogenesis is through changes in the composition of the intestinal microbiota, resulting in bacterial imbalance. Broad-spectrum antibiotics such as fluoroquinolones can reduce intestinal biodiversity by affecting the composition of the intestinal microbiota, and even have an impact on intestinal homeostasis for a long period of time after exposure to antibiotics. Animal study confirmed that the proportion of Firmicutes and Bacteroidetes phyla/species in obese mice was higher than that in non-obese mice (24). A high value of evidence indicated that changes in Firmicutes and Bacteroidetes phyla/species levels might be significant indicators/factors for obesity in children (25). Antibiotic exposure may affect the production of short-chain fatty acids (SCFAs) by altering bacterial abundance. SCFAs serve as energy substrates to provide energy to the host and as signal regulators and participate in the regulation of lipids (26). In addition, the intestinal microbiota can influence host energy metabolism by affecting bile acid metabolism and its signaling pathways (27). Animal experiments have confirmed that continued exposure to low-dose antibiotics can significantly increase body fat content in mice (28). As early as the 1950s, antibiotics were found to promote weight gain in aquatic animals such as livestock and poultry. Therefore, low-dose antibiotics have long been used as feed additives to enhance animal growth. Studies have indicated that late application of antibiotics, compared to earlier applications, can increase animal body weight (29), providing further evidence for the growth-promoting effects of VAs and PVAs.

Our findings revealed that exposure to fluoroquinolones, particularly norfloxacin and tetracyclines was associated with overweight/obesity in boys. Fluoroquinolones are usually used to treat and prevent diseases in animals. They also represent the most commonly detected class of antibiotics in aquatic products, milk, livestock, and poultry, often detected at relatively high concentrations (30–32). Tetracyclines, on the other hand, are the most commonly used antibiotics in animal husbandry, and both fluoroquinolones and tetracyclines are classified as *β*-diketone antibiotics (33). Previous studies have reported that exposure to β-diketone antibiotics may lead to obesity in the older adult (34). In an animal experiment, zebrafish exposed to high concentrations of β-diketone antibiotics exhibited higher body weight and BMI compared to controls, with male zebrafish displaying higher body weights across all concentrations of β-diketone antibiotics (35). Moreover, animal experiments have shown that the use of fluoroquinolones disrupts the type and quantity of the intestinal microbiota in mice (36).

Notably, our findings revealed that exposure to lower concentrations of oxytetracycline and tetracycline was associated with overweight and obesity in children, whereas exposure to higher concentrations did not. An animal study found that exposure to low doses of penicillin or oxytetracycline caused weight gain in mice, whereas high doses resulted in weight loss (37). This phenomenon may be due to the varied effects of antibiotic exposure on body weight at different doses. Therefore, further research is needed to explore the relationship between different types and concentrations of antibiotic exposure and overweight/obesity.

Our study revealed sex-specific differences in the association between antibiotic exposure and overweight/obesity. Sulfaclozine, oxytetracycline, norfloxacin, tetracyclines, and quinolones were independently and positively associated with the risk of overweight/obesity in boys. Moreover, higher levels of *β*-lactams were significantly correlated with the risk of overweight/obesity in girls. This sex-specific correlation may be attributed to several factors. First, there are obvious differences in exposure levels to different antibiotics among children of different sexes (16). Second, disparities in dietary and behavioral habits between boys and girls (38) could affect the association between antibiotic exposure and obesity. Finally, the levels of sex hormones are different in boys and girls, and some studies have indicated that sex hormone levels influence the structure and function of intestinal flora (39, 40). Therefore, the sex-specific association between antibiotic exposure and childhood overweight/obesity may be related to the sex-specific gut microbiota.

Furthermore, our findings revealed that exposure to several antibiotics, such as ulfaclozine, oxytetracycline, and norfloxacin, was associated with childhood overweight/obesity. Moreover, a cross-sectional study examining the relationship between antibiotics and obesity in children in Shanghai, China, revealed that exposure to florfenicol and trimethoprim was positively correlated with overweight/obesity in children aged 8–12 years (15). In a case–control study conducted in Shandong and Guangdong provinces in China exposure to florfenicol was found to be associated with overweight/obesity in children aged 6–9 years (16). Another cross-sectional study from northern China indicated that exposure to enrofloxacin was associated with an increased risk of overweight/obesity in children aged 0–15 years (12). Several reasons may account for the observed inconsistent results. First, variations in environmental conditions and dietary habits of populations across different research areas can result in different antibiotic detection rates. In addition, the lower detection rates of some antibiotics (such as florfenicol and trimethoprim) suggest their exclusion from the association analysis.

This study is not without its limitations. Firstly, the study only investigated dietary frequency and not dietary intake. A retrospective collection of children’s dietary consumption data conducted through a questionnaire survey may have introduced recall bias. Secondly, as a cross-sectional study, it did not establish a causal relationship between antibiotic exposure and overweight/obesity. Finally, most antibiotics have short half-lives, which may not adequately represent long-term exposure levels to antibiotics.

## 5 Conclusion

Using BMI as an indicator of overweight/obesity and employing a biomonitoring approach, our findings reveal that exposure to foodborne antibiotics (VAs and PVAs) in children increases the risk of overweight/obesity and highlights sex disparities in the association between antibiotic exposure and overweight/obesity. It is necessary to further regulate the use of antibiotics in animal husbandry and aquaculture, enhance the monitoring of antibiotic residues in animal-derived foods, and maintain a focus on children’s exposure to foodborne antibiotics.

## Data Availability

The original contributions presented in the study are included in the article/Supplementary material, further inquiries can be directed to the corresponding authors.
