# Supplementary material for: Antibiotic exposure and risk of overweight/obesity in children: a biomonitoring-based study from eastern Jiangsu, China
Source: Front Public Health. 2024 Nov 8;12:1494511. doi: 10.3389/fpubh.2024.1494511 (PMC11582042; doi:10.3389/fpubh.2024.1494511)
Supplement: Supplementary file 1 [file Table_1.pdf]

Supplementary Material

1 Supplementary Tables

Table S1. Usage, molecular formula, molecular weight, and CAS number of the target antibiotics

Compound	Usage	Molecular formula	Molecular weight	CAS number
**Sulfonamides**				
Sulfamethoxazole	PVA	C10H11N3O3S	253.28	723-46-6
Sulfameter	PVA	C11H12N4O3S	280.30	651-06-9
Sulfaquinoxaline	VA	C14H12N4O2S	300.34	59-40-5
Sulfaclozine	VA	C10H9ClN4O2S	284.72	102-65-8
Trimethoprim	PVA	C14H18N4O3	290.32	738-70-5
Sulfamerazine	PVA	C11H12N4O2S	264.3 0	127-79-7
Sulfamerazine	PVA	C10H10N4O2S	250.28	68-35-9
sulfachloropyridazine	VA	C10H9ClLN4O2S	284.72	80-32-0
Sulfamonomethoxine	VA	C11H12N4O3S	280.30	1220-83-3
**Macrolides**				
Erythromycin	PHA	C37H67NO13	733.94	114-07-8
Clarithromycin	HA	C38H69NO13	747.95	81103-11-9
Azithromycin	HA	C38H72N2O12	748.98	83905-01-5
Roxithromycin	HA	C41H76N2O15	837.05	80214-83-1
Tilmicosin	VA	C46H80N2O13	869.13	108050-54-0
Tylosin	VA	C46H77NO17	916.1 0	1401-69-0
**β -lactams**				
Ceftiofur	VA	C19H17N5O7S3	523.56	80370-57-6
Cefquinome	VA	C23H24N6O5S2	528.60	84957-30-2
Ampicillin	PHA	C16H19N3O4S	349.4 1	69-53-4
cefotaxime	HA	C16H16N5NaO7S2	477.45	64485-93-4
Penicillin V	PHA		455.4 7	63527-52-6
Penicillin V	PHA	C16H18N2O5S	350.39	1987/8/1
Amoxicillin	PHA	C16H19N3O5S	365.40	26787-78-0
Cefdinir	HA	C14H13N5O5S2	395.41	91832-40-5
**Tetracyclines**				
Oxytetracycline	PVA	C22H24N2O9	460.43	79-57-2
Chlorotetracycline	PVA	C22H23CLN2O8	478.88	57-62-5
Tetracycline	PVA	C22H24N2O8	444.44	60-54-8
Deoxytetracycline	PVA	C22H24N2O8	444.44	564-25-0
**Quinolones**				

Pefloxacin	PVA	C17H20FN3O3	333.36	70458-92-3
Lomefloxacin	PVA	C17H19F2N3O3	351.35	98079-51-7
Danofloxacine	VA	C18H22FN3O3	357.38	112398-08-0
Sarafloxacin	VA	C20H17F2N3O3	385.36	98105-99-8
Ofloxacin	PVA	C18H20FN3O4	361.37	82419-36-1
Difloxacin	VA	C21H19F2N3O3	399.39	98106-17-3
Enrofloxacin	VA	C19H22FN3O3	359.40	93106-60-6
Ciprofloxacin	PVA	C17H18FN3O3	331.35	85721-33-1
Norfloxacin	PVA	C16H18FN3O3	319.33	70458-96-7
**Quinoxalines**				
Quinocetone	VA	C18H14N2O3	306.3 2	81810-66-4
**Lincosamides**				
Lincomycin	PVA	C18H34N2O6S	406.54	154-21-2
**Chloramphenicols**				
Chloramphenicol	HA	C11H12CL2N2O5	323.14	56-75-7
Thiamphenicol	PVA	C12H15CL2NO5S	356.22	15318-45-3
Florfenicol	VA	C12H14ClL2FNO4S	358.22	73231-34-2

HAs:human antibiotics, VAs:veterinary antibiotics, PHAs:preferred human antibiotics, PVAs:preferred veterinary antibiotics
